# Entwicklung und Validierung eines Fragebogens zur Erfassung postural-strukturell-biomechanisch orientierter Überzeugungen von Physiotherapeut:innen zu Schmerzen

**DOI:** 10.1007/s00482-023-00757-y

**Published:** 2023-10-16

**Authors:** Ahura Bassimtabar, Martin Alfuth

**Affiliations:** 1 Fachbereich Gesundheitswesen, Hochschule Niederrhein, Reinarzstr. 49, 47805 Krefeld, Deutschland; 2 Universitäre Weiterbildung M.Sc. Sportphysiotherapie, Deutsche Sporthochschule Köln, Am Sportpark Müngersdorf 6, 50933 Köln, Deutschland; 3 Bayer 04 Leverkusen Fußball GmbH, Bismarckstr. 122–124, 51373 Leverkusen, Deutschland

**Keywords:** Bio-psycho-sozial, Kompetenz, Messung, Evaluation, Evidenz, Bio-psycho-social, Competence, Measurement, Evaluation, Evidence

## Abstract

**Hintergrund:**

Physiotherapeut:innen verwenden in der klinischen Praxis häufig das postural-strukturell-biomechanische (PSB) Modell, um das Symptom Schmerz mit biomechanischen Defiziten zu erklären. Ein adäquates Wissen über Schmerz umfasst jedoch nicht nur die Kenntnis über die Neurophysiologie des Schmerzes, sondern auch die Kenntnis, dass allein PSB-orientierte Erklärungen über die Entstehung und Verstärkung von Schmerzen veraltet sind. Ein Fragebogen zur Erfassung von PSB-orientierten Überzeugungen existiert bisher nicht.

**Ziel:**

Ziel dieser Studie war, einen neuen Fragebogen zur Erfassung der postural-strukturell-biomechanisch orientierten Überzeugungen von Physiotherapeut:innen zu Schmerzen zu konzipieren und hinsichtlich seiner Reliabilität (interne Konsistenz), Validität und Übereinstimmung zu prüfen.

**Methoden:**

Der Essential Knowledge of Pain Questionnaire (EKPQ) wurde auf Grundlage einer Literaturrecherche und von Diskussionen zwischen Experten konstruiert. Anschließend wurde eine Pilotstudie durchgeführt, in der 32 Physiotherapieschüler:innen aus einer Fachschule für Physiotherapie online (SoSci Survey) mittels der deutschen Version des Revised Neurophysiology of Pain Questionnaire (rNPQ-D) und des EKPQ hinsichtlich ihres Wissens über und ihrer Überzeugungen zu Schmerzen befragt wurden.

**Ergebnisse:**

Die interne Konsistenz des EKPQ war mit Cronbach’s α = 0,784 akzeptabel. Es bestand eine positive signifikante Korrelation von r = 0,518 (*p* = 0,002) zwischen den beiden Fragebögen. Die Bland-Altman-Analyse ergab eine mittlere Differenz zwischen den Fragebögen von 28,9 (± Standardabweichung der Differenz 15,3) % mit einer oberen Grenze der 95 %-Übereinstimmung von 58,8 % und einer unteren Grenze der 95 %-Übereinstimmung von −1,0 %. Im rNPQ‑D erzielten die Teilnehmer:innen einen Score von im Mittel 60,7 %, im EKPQ einen Score von im Mittel 31,8 %.

**Schlussfolgerung:**

Der neu konstruierte Fragebogen EKPQ scheint ein reliables und valides Instrument zu sein, um PSB-orientierte Überzeugungen von Therapeut:innen zu Schmerzen zu erfassen. Die Ergebnisse zeigen darüber hinaus, dass ein hohes Wissen über Schmerzneurophysiologie im rNPQ eine PSB-Orientierung nicht ausschließt. Ob der EKPQ neben dem rNPQ als zusätzliches Assessment zur Überprüfung von Überzeugungen zu Schmerzen dienen kann, sollte zukünftig mit passenden Studiendesigns, z. B. Delphi-Studie, untersucht werden.

**Graphic abstract:**

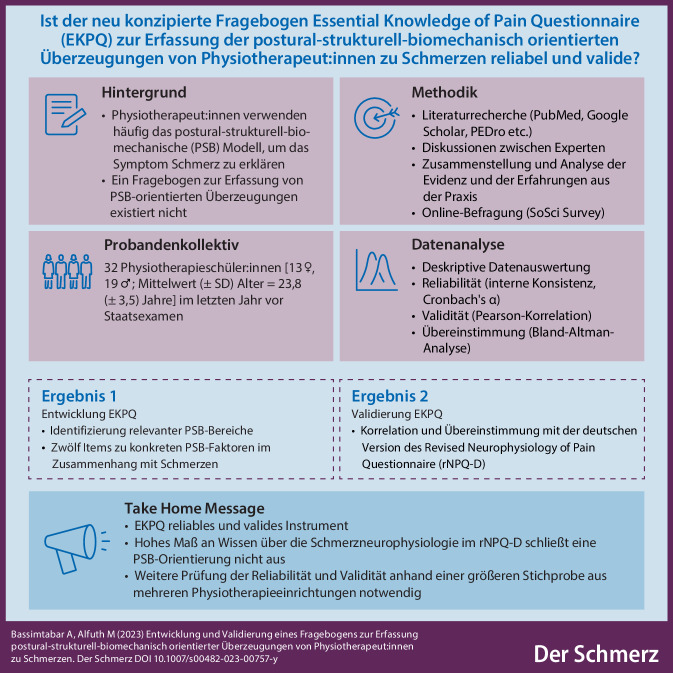

## Abstract (en)

**Background:**

Therapists frequently use the postural-structural-biomechanical (PSB) model in clinical practice to explain the symptom of pain using biomechanical deficits. Adequate knowledge about pain encompasses not only the neurophysiology of pain but also knowledge that existing PSB-oriented explanations of the development and enhancement of pain are outdated. There is no assessment to evaluate physiotherapists’ PSB-oriented beliefs about pain.

**Aim:**

The aim of the present study was to develop a questionnaire to assess physiotherapists’ PSB-oriented beliefs about pain and to evaluate its reliability (internal consistency), validity, and agreement.

**Methods:**

The Essential Knowledge of Pain Questionnaire (EKPQ) was constructed on the basis of a literature search and discussions between experts. In a pilot study, 32 pupils of a physiotherapy school were then asked to complete the German version of the revised Neurophysiology of Pain Questionnaire (rNPQ-D) and the EKPQ using the SoSci Survey in order to assess their knowledge and beliefs about pain.

**Results:**

The internal consistency of the EKPQ was acceptable with a Cronbach’s α = 0.784. There was a strong positive significant correlation between the questionnaires (r = 0.518; *p* = 0.002). The Bland–Altman analysis revealed a mean difference of 28.9% (± standard deviation of the difference 15.3%) with an upper limit of 95% agreement of 58.8% and a lower limit of 95% agreement of −1.0% between the questionnaires. Participants achieved a mean score of 60.7% in the rNPQ‑D and a mean score of 31.8% in the EKPQ.

**Conclusion:**

The newly developed EKPQ questionnaire seems to be a reliable and valid assessment to determine physiotherapists’ PSB-oriented beliefs about pain. The results also confirm that a high level of knowledge about the neurophysiology of pain does not exclude a PSB orientation. Whether the EKPQ can be used alongside the rNPQ as an additional assessment to evaluate beliefs about pain should be investigated in the future with suitable study designs, e.g. Delphi study.

## Einleitung

Das biomedizinische Modell des Schmerzes wurde Ende der 1970er-Jahre um psychische und soziale Faktoren erweitert [8] und hat sich mittlerweile als biopsychosoziales Modell in der Wissenschaft etabliert. Die neueste und überarbeitete Version der International Association for the Study of Pain definiert Schmerz als eine unangenehme sensorische und emotionale Erfahrung, die mit einer tatsächlichen oder potenziellen Gewebeschädigung in Zusammenhang steht oder einer solchen ähnelt [18]. Häufig sind beispielsweise Kreuzschmerzen unspezifisch, was bedeutet, dass den Schmerzen keine erklärbare strukturelle Schädigung oder Pathologie zugrunde liegt [1, 11, 17]. Sowohl Schmerzfreiheit bei radiologisch nachweisbaren strukturellen Schädigungen als auch Schmerzpersistenz bei radiologischer Unauffälligkeit unterstreichen die multifaktoriellen Ursachen von Schmerzen [2]. Schmerz wird somit nicht nur mit mechanisch-strukturellen Faktoren in Verbindung gebracht, sondern auch mit nichtmechanischen, affektiven, kognitiven, emotionalen und sozialen Faktoren [15], die in der Physiotherapie Berücksichtigung finden sollten [14, 26].

Entgegen der Erkenntnisse aus der Neurophysiologie und Schmerzwissenschaft verwenden Physiotherapeut:innen häufig das postural-strukturell-biomechanische (PSB) Modell, um das Symptom Schmerz mit biomechanischen Defiziten zu erklären, und sind überzeugt, dass muskuloskelettale Asymmetrien und Dysbalancen zu Schmerzen führen [10]. Ein biomechanisches Defizit ist beispielsweise eine „schlechte“ Haltung, die mit einer Hyperkyphose der Brustwirbelsäule und einer anterioren Positionierung der Halswirbelsäule assoziiert ist. Es besteht jedoch kein Kausalzusammenhang zwischen vermeintlich schlechter Haltung und Schmerz [21]. Weitere Beispiele für biomechanische Defizite sind eine ausgeprägte Lendenlordose, ein erhöhter Knievalguswinkel, Beinlängendifferenzen, Beckenschiefstände, defizitäre neuromuskuläre Ansteuerung, wie etwa die der Mm. multifidi oder des M. transversus abdominis, oder vermeintlich falsche Hebetechniken von Lasten mit rundem Rücken. Jedoch stehen Schmerzen und Schmerzursache in keinem Zusammenhang mit PSB-Faktoren [10]. Für das therapeutische Verständnis von Schmerzen und deren Entstehung scheint demnach wichtig zu sein, nicht nur die Relevanz der Neurophysiologie und der psychosozialen Faktoren herauszustellen, sondern auch die häufig bestehende Fokussierung auf biomechanische Defizite zu reduzieren. In einer Studie aus dem Jahr 2003 wurde bekannt, dass das Gesundheitspersonal zu wenig über Schmerzen weiß [13]. So beantworteten die Teilnehmer:innen, zu denen Physiotherapeut:innen, Ergotherapeut:innen, Ärzte und Ärztinnen sowie Psycholog:innen gehörten, im Neurophysiology of Pain Questionnaire (NPQ) nur 55 % der Fragen richtig. Der NPQ dient zur Erhebung des Wissensstands über die Neurophysiologie von Schmerzen und wurde zur Verbesserung der psychometrischen Eigenschaften überarbeitet und als Revised Neurophysiology of Pain Questionnaire (rNPQ) bezeichnet [3, 16]. Einige essenzielle Bereiche der Schmerzwissenschaft, welche insbesondere PSB-Faktoren beinhalten, werden jedoch nicht hinreichend berücksichtigt. Es besteht aber die Vermutung, dass ein hohes Maß an Wissen des Behandlers/der Behandlerin über die Schmerzneurophysiologie nicht gleichzeitig eine PSB-orientierte Betrachtung der Schmerzen von Patient:innen ausschließt, was möglicherweise zu Fehlinterpretationen und Fehlbehandlungen führen kann. Um auch PSB-Faktoren in der Überprüfung der Überzeugungen zu Schmerzen zu berücksichtigen, war das Ziel dieser Studie, einen neuen Fragebogen, den Essential Knowledge of Pain Questionnaire (EKPQ), zu konzipieren und hinsichtlich seiner Reliabilität (interne Konsistenz), Validität und Übereinstimmung zu prüfen.

## Methodik

### Entwicklung des EKPQ

In dieser Studie wurde ein neuer Fragebogen (EKPQ) entwickelt, der den evidenzbasierten Wissensstand und die Überzeugungen zu PSB-Faktoren im Zusammenhang mit Schmerzen erfassen soll. Im Rahmen der Konstruktion wurden die Reliabilität (interne Konsistenz), Validität und Übereinstimmung des EKPQ bestimmt.

### Inhaltsvalidität

Um die Inhaltsvalidität des Fragebogens zu gewährleisten, wurde zunächst die Literatur gesichtet. Relevante Studien zum Thema Schmerzen wurden daraufhin einbezogen. Weiter wurde zur Orientierung der rNPQ herangezogen. Der rNPQ besteht aus zwölf Items, abgeleitet vom NPQ, der ursprünglich 19 Items beinhaltet [13]. Die Konstruktvalidität und Test-Retest-Reliabilität des rNPQ werden als akzeptabel beschrieben [3]. Die interne Konsistenz der deutschen Version des rNPQ (rNPQ-D) ist mit Cronbach’s alpha (α) = 0,61 bei der Anwendung bei Therapeut:innen [19] als fragwürdig zu interpretieren. Im rNPQ‑D werden zwölf verschiedene Aussagen mit je drei Antwortmöglichkeiten („richtig“, „falsch“ oder „unentschlossen“) vorgegeben, die von der Versuchsperson ausgewählt werden sollen [12]. Für jede richtig gegebene Antwort wird ein Punkt vergeben, sodass der maximale Score zwölf Punkte beträgt. Für eine falsche Antwort oder die Antwort „unentschlossen“ gibt es keinen Punkt. Je höher der Score, desto höher ist der Wissensstand über Schmerz einzuordnen. Der Score stellt abschließend in Prozent dar, wie hoch der Anteil der richtigen Antworten ist. Die Anzahl der richtigen Antworten wird zur Berechnung durch die Anzahl der Fragen (12) dividiert und mit 100 multipliziert.

Es fanden darüber hinaus regelmäßig Diskussionen zwischen Experten, zwei Physiotherapeuten und einem Arzt statt, in denen die Evidenz aus der Literatur und die Erfahrungen aus der Praxis zusammengetragen und analysiert wurden. Hierbei wurden folgende relevante PSB-Bereiche identifiziert: Diagnostik, Haltung, Aktivität, Intervention und Risikofaktoren. Zu diesen Bereichen wurden insgesamt zwölf Items zu konkreten PSB-Faktoren in Zusammenhang mit Schmerz formuliert und in eine Reihenfolge gebracht. Weiter wurden acht Dummy-Items mit dem Fokus auf die Anatomie der Wirbelsäule konstruiert, um kognitive Verzerrungen des Antwortverhaltens, wie z. B. „confirmation bias“, „overconfidence bias“, „recall bias“ oder soziale Erwünschtheit, zu minimieren. Diese Items flossen nicht in den Score ein. Zwei weitere Items zur Therapie von akuten und chronischen Rückenschmerzen dienten nur zur Diskussion, da hier keine eindeutig richtige oder falsche Antwort gültig war. Diese wurden ebenso nicht im Score berücksichtigt. Wie beim rNPQ entstand der Score beim EKPQ aus der Beantwortung von 12 Items. Je höher der Score im rNPQ ist, umso höher ist das Wissen der Therapeut:innen über die Neurophysiologie des Schmerzes. Je höher der Score im EKPQ ist, umso weniger PSB-orientierte Überzeugungen in Bezug auf Schmerz hat der/die Therapeut:in. Idealerweise erreichen Physiotherapeut:innen somit möglichst hohe Scores in beiden Fragebögen.

Anschließend wurde eine Pilotstudie durchgeführt, in der sowohl der rNPQ‑D als auch der EKPQ bei einer Gruppe von Physiotherapieschüler:innen (*n* = 32) aus einer Fachschule für Physiotherapie, die sich im letzten Ausbildungsjahr befanden, angewendet wurde. Es wurde ein Online-Link auf der SoSci-Survey-Plattform konzipiert, der den rNPQ‑D und EKPQ beinhaltete, welcher den Schüler:innen zur Verfügung gestellt wurde. Beim Ausfüllen der Fragebögen wurden die Schüler:innen online supervisiert, um die Bedingungen bei der Beantwortung konstant zu halten und einen möglichen Antwort- und Stichprobenausfall („non-response bias“) zu minimieren [20].

### Datenreduktion und statistische Analyse

Für eine richtige Antwort wurde der Wert 1, für eine falsche Antwort oder die Antwort „unentschlossen“ der Wert 0 vergeben. Die Anzahl der Punkte wurde zu einem Gesamtwert addiert. Dieser Gesamtwert wurde durch die Anzahl der Items (12) dividiert und mit 100 multipliziert, um den Score in Prozent (%) zu erhalten. Die Scores (%) der Teilnehmer:innen wurden gemittelt. Zudem wurden die Standardabweichungen (±SD) und 95 %-Konfidenzintervalle (95 %-KI) bestimmt. Es wurde die Reliabilität (interne Konsistenz) des EKPQ (zwölf Items) mit Cronbach’s α, dem Maß für die Verbundenheit von Items in einem Fragebogen [5, 23, 24], bestimmt. Ein Wert von Cronbach’s α ≥ 0,7 gilt gewöhnlich als akzeptabler Standard der internen Konsistenz. Zur Bestimmung der Konstruktvalidität wurde die Korrelation zwischen den beiden Fragebögen mit dem Korrelationskoeffizienten nach Pearson (r) berechnet. Ein r = 0,1 gilt als schwache, ein r = 0,3 als moderate und ein r = 0,5 als starke Korrelation [4]. Das Signifikanzniveau wurde auf α = 0,05 festgelegt. Zur Beurteilung der Übereinstimmung der Scores (%) der beiden Fragebögen wurde eine Bland-Altman-Analyse mit Bestimmung der 95 %-Übereinstimmungsgrenzen durchgeführt [9]. Boden- und Deckeneffekte des EKPQ-Fragebogens wurden unter Verwendung der Summenwerte analysiert. Wenn weniger als 15 % der Teilnehmer:innen den maximalen (12) bzw. den minimalen Wert (0) erreichen, weist der Fragebogen keine Boden- und Deckeneffekte auf [24]. Die statistischen Analysen wurden mittels Microsoft Excel (2016; Microsoft Corporation, Redmond, WA, USA) sowie Statistical Package for Social Sciences (SPSS Statistics, Version 28.0; IBM Corporation, Armonk, NY, USA) durchgeführt.

## Ergebnisse

Alle Items des EKPQ-Fragebogens (22) sind in den Tab. 1 und 2 dargestellt. Die zwölf Items, die den Score bilden, werden mit den entsprechenden Antworten in Tab. 1 aufgeführt. Die acht Dummy-Items und die beiden Diskussionsitems befinden sich in Tab. 2. Die Teilnehmer:innen – 13 weibliche und 19 männliche – waren im Mittel 23,78 (±3,48) Jahre alt.

**Tab. 1:** Die zwölf zu wertenden Items (2, 3, 5, 6, 8, 11, 12, 14, 18, 19, 20, 21) des Essential Knowledge of Pain Questionnaire (EKPQ). Die richtigen Antworten wurden hinter jedem entsprechenden Item mit einer Raute gekennzeichnet. Die acht Dummy-Items und die zwei Diskussionsitems wurden entsprechend ausgeblendet

Item	Formulierung	Richtig	Falsch
2	Ein in der bildgebenden Diagnostik deutlich sichtbarer Bandscheibenvorfall kann sich ohne operative, invasive oder konservative Behandlung spontan und vollständig zurückbilden	#	–
3	Bandscheibenvorfälle mit Austritt des Nucleus pulposus verursachen nicht zwingend Schmerzen	#	–
5	DIM und SIM stehen für:	Danger in me; safety in me	Dynamic inhibited muscle; static inhibited muscle
6	Folgender Haltungstyp führt eher zu Nackenschmerzen:	Keiner	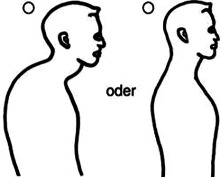
8	Manuelle Therapie (z. B. Traktion/Translation) ist grundsätzlich effektiver in Bezug auf Schmerzlinderung als eine Scheintherapie	–	#
11	Folgende Beinachse führt beim Laufen eher zu Knieschmerzen	Keine	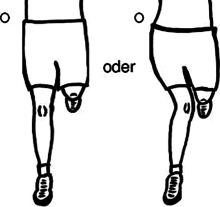
12	Bei therapeutischen Übungen (z. B. bei Patienten mit Kniearthrose) ist es unter Umständen vollkommen in Ordnung, in den Schmerz „reinzutrainieren“	#	–
14	Es besteht ein Zusammenhang zwischen Beckenschiefstand und Rückenschmerzen	–	#
18	Folgenden Hebetyp sollte man bei Rückenschmerzen vermeiden:	Keinen	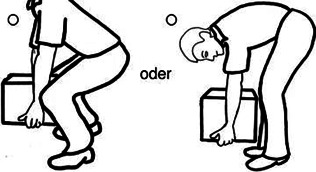
19	Mittels bildgebender Verfahren (z. B. MRT) kann eine Schmerzursache zuverlässig diagnostiziert werden	–	#
20	Bei der Behandlung von unspezifischen Rückenschmerzen ist die Kräftigung der tiefen Rumpfmuskeln wichtiger als die der oberflächlichen	–	#
21	Nocebos sind in der Therapie ein Risikofaktor für Chronifizierung und Katastrophisierung	#	–

*DIM* Danger in me, *SIM* Safety in me, *MRT* Magnetresonanztomographie

**Tab. 2:** Die acht Dummy-Items (1, 4, 7, 9, 10, 15, 16, 22) und die zwei Diskussionsitems (13, 17)

Item	Formulierung	Antwortmöglichkeiten	
1	Wählen Sie die richtige Zahlenkombination mit folgender Reihenfolge: Nucleus pulposus, Anulus fibrosus, Spinalnerv	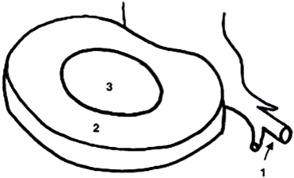	O 2, 3, 1 O 1, 3, 2 O 3, 2, 1
4	In der Abbildung sind die HWS-Extensoren auf Länge, die Flexoren in Annäherung	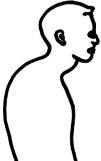	O Richtig O Falsch O Unentschlossen
7	Nennen Sie die Maximal-loose-packed-Position (MLPP) der Schulter	–	
9	Nach dem Kaltenborn-Prinzip gleitet der Humeruskopf bei Schulterabduktion nach dorsal	O Richtig O Falsch O Unentschlossen	
10	Wie verläuft das Lig. collaterale mediale (Knieinnenband)	Verläuft von: bis: ggf. Besonderheit:	
13	Welche der angeführten Interventionen halten Sie bei chronischen Schmerzpatienten als „first line treatment“ (Beginn einer Therapie) für angemessen?	O Edukation O Bewegungstherapie O Manuelle Therapie (MT) O Akupunktur O Progressives Krafttraining O Massage	
15	Bei dieser Patientin ist die LWS nach rechts lateralflektiert	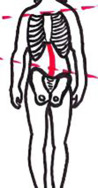	O Richtig O Falsch O Unentschlossen
16	Bei dieser Übung („cat/cow“) wird die Wirbelsäule in der Sagittalebene mobilisiert	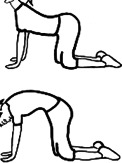	O Richtig O Falsch O Unentschlossen
17	Was empfehlen Sie einem Patienten, der seit zwei Tagen Rückenschmerzen lumbal rechtsseitig bei LWS-Flexion hat? (es liegen keine „red flags“ vor)	O Beraten und abwarten („wait and see“) O Eine Form von aktiver Therapie O Eine Form von passiver Therapie	
22	Bei dieser Übung wird die Patientin dazu angeleitet, ihren Bauchnabel leicht einzuziehen. Dabei werden die Mm. multifidi aktiviert	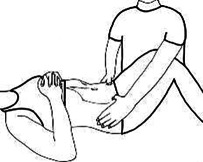	O Richtig O Falsch O Unentschlossen

*HWS* Halswirbelsäule, *MLPP* Maximal-loose-packed-Position, *Lig.* Ligamentum, *MT* Manuelle Therapie, *LWS* Lendenwirbelsäule, *Mm.* Musculi

### Reliabilität (interne Konsistenz), Konstruktvalidität und Übereinstimmung des EKPQ

**Abb. 1:**
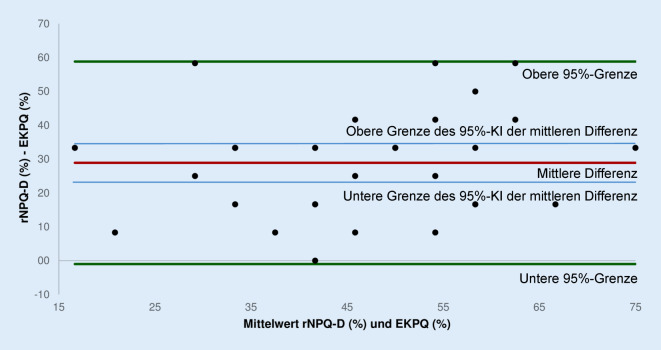
Bland-Altman-Plot zur Analyse des Grades der Übereinstimmung zwischen dem Essential Knowledge of Pain Questionnaire (EKPQ) und der deutschen Version des Revised Neurophysiology of Pain Questionnaire (rNPQ-D) mit den Angaben mittlere Differenz (*rote Linie*), 95 %-Konfidenzintervall (KI) der mittleren Differenz (hellblaue Linien) und 95 %-Grenzen der Übereinstimmung (*grüne Linien*)

Für die interne Konsistenz des EKPQ wurde ein Wert von Cronbach’s α = 0,784 berechnet. Die Item-Skala-Statistiken sind in Tab. 3 aufgeführt. Im rNPQ‑D erzielten die Teilnehmer:innen von 12 möglichen Punkten im Mittel 7,3 ± 2,0 Punkte. Das entsprach einem Score von im Mittel 60,7 ± 16,6 (95 %-KI 54,9 bis 66,4) %. Im EKPQ hingegen betrug die erreichte Punktzahl im Mittel 3,8 ± 1,7 Punkte von 12 möglichen Punkten, was einem Score von im Mittel 31,8 ± 14,3 (95 %-KI 26,8 bis 36,7) % entsprach. Es bestand eine positive signifikante Korrelation von r = 0,518 (*p* = 0,002) zwischen den beiden Fragebögen. Die Bland-Altman-Analyse ergab eine mittlere Differenz zwischen den Fragebögen von 28,9 (± Standardabweichung der Differenz 15,3) % mit einer oberen Grenze der 95 %-Übereinstimmung von 58,8 % und einer unteren Grenze der 95 %-Übereinstimmung von −1,0 % (Abb. 1). Die obere Grenze des 95 %-Konfidenzintervalls (KI) der mittleren Differenz betrug 34,4 %, die untere Grenze des 95 %-KIs der mittleren Differenz 23,4 %.

**Tab. 3:** Essential Knowledge of Pain Questionnaire (EKPQ), Item-Skala-Statistiken der 12 gewerteten Items

Nummer EKPQ-Item (gewertet)	Skalenmittelwert, wenn Item weggelassen	Skalenvarianz, wenn Item weggelassen	Korrigierte Item-Skala-Korrelation	Cronbach’s α, wenn Item weggelassen
Item 2	18,0909	20,710	0,500	0,760
Item 3	18,1212	21,485	0,393	0,773
Item 5	17,6364	24,426	0,122	0,794
Item 6	18,5758	24,002	0,322	0,778
Item 8	18,0909	21,773	0,384	0,773
Item 11	18,4242	23,377	0,413	0,772
Item 12	18,0909	20,273	0,532	0,756
Item 14	18,1818	20,466	0,609	0,748
Item 18	18,6061	22,621	0,682	0,758
Item 19	18,4545	21,818	0,466	0,764
Item 20	17,7576	23,064	0,197	0,796
Item 21	18,3030	19,530	0,661	0,739

### Boden- und Deckeneffekte

Der EKPQ zeigte weder Boden- noch Deckeneffekte. Das Minimum im Fragebogen lag bei einem Wert von 0 (*n* = 1; 3,13 %), das Maximum bei 7 (*n* = 2; 6,25 %).

## Diskussion

Ziel dieser Studie war die Entwicklung eines neuen Fragebogens, mit Prüfung der internen Konsistenz, Validität und Übereinstimmung, der die PSB-orientierten Überzeugungen von Physiotherapeut:innen zu Schmerzen beurteilen soll. Der Fragebogen soll den rNPQ‑D nicht ersetzen, sondern sinnvoll ergänzen. Die wichtigste Erkenntnis der vorliegenden Studie war, dass der EKPQ ein reliabler und valider Fragebogen im Vergleich zur Referenz, dem rNPQ‑D, zu sein scheint. Das zeigen die akzeptable interne Konsistenz des EKPQ mit Cronbach’s α = 0,784, welche höher ist als die des rNPQ‑D (Cronbach’s α = 0,61; [19]), sowie die positive Korrelation (r = 0,518) und Übereinstimmung zwischen dem EKPQ und dem rNPQ‑D.

### Wissenschaftliche Einordnung der Ergebnisse

Die Validität des EKPQ lässt sich nicht unmittelbar mit Angaben zum rNPQ‑D und zu der französischen Version des NPQ (NPQ-Fr) vergleichen, da hinsichtlich dieser die diskriminante Validität bestimmt wurde. Zum einen wurden Korrelationen zwischen dem NPQ-Fr und ausgewählten Scores des Fragebogens Short Form 36 (SF-36) berechnet [7]. Diese zeigten, dass die Fragebögen verschiedene Konstrukte messen. Zum anderen attestierten schwache Korrelationen zwischen dem rNPQ‑D und den Fragebögen Short Form 12 (SF-12) sowie Funktionsfragebogen Hannover - Rücken (FFbH‑R) der deutschen Version ebenfalls die Konstruktvalidität [19].

Obwohl sich insgesamt eine gute Übereinstimmung zwischen dem EKPQ und dem rNPQ‑D in der Bland-Altman-Analyse zeigt, ist der Grad der Übereinstimmung zwischen den Fragebögen nicht signifikant, da die Nulllinie (Übereinstimmung zwischen den Messmethoden) unterhalb des Konfidenzintervalls der mittleren Differenz liegt [9]. Das bedeutet, dass die Überzeugungen zu Schmerzen mittels des EKPQ im Vergleich zum Wissen mittels des rNPQ‑D möglicherweise unterschätzt werden. Untermauert wird diese Annahme dadurch, dass die Teilnehmer:innen im EKPQ um 28,9 % schlechter abschnitten als im rNPQ‑D.

### *Klinische Relevanz der Ergebnisse*

Um Patient:innen mit anhaltenden Schmerzen adäquat zu versorgen, ist die richtige Interpretation der Schmerzentstehung essenziell. Vergangene Studien zeigten jedoch, dass Therapeut:innen hier ein defizitäres Wissen offenbaren [7, 13]. Auf die Praxis des Schmerzmanagements kann sich dieses defizitäre Wissen nachteilig auswirken [19]. Mit r = 0,518 gab es eine positive Korrelation zwischen den Scores beider Fragebögen, sodass womöglich ausschließlich neurophysiologische Lehre (Verbesserung des rNPQ-Scores) PSB-orientierte Überzeugungen reduziert (Verbesserung des EKPQ-Scores). Dies müsste in zukünftigen Studien untersucht werden. Dennoch fielen trotz der positiven Korrelation die Scores des EKPQ deutlich niedriger aus als die Scores im rNPQ. Dies verdeutlicht, dass ein hoher Wissensstand in der Neurophysiologie (hoher Score im rNPQ) eine PSB-Orientierung (niedriger Score im EKPQ) nicht ausschließt, sodass der Einsatz des EKPQ als zusätzlicher Fragebogen, welcher PSB-orientierte Überzeugungen des Therapeuten in Bezug auf Schmerz quantifiziert, sinnvoll wäre.

Nach Erfahrungen wird an Fachschulen für Physiotherapie in Deutschland häufig noch gelehrt, dass Schmerz Nozizeption sei. Dass häufig chronische Schmerzen mit mangelnder peripherer Gewebeheilung assoziiert werden, ist darüber hinaus als ein bedeutendes Missverständnis von Schmerzen zu bewerten. Im EKPQ werden PSB-Faktoren aus den Bereichen Diagnostik, Haltung, Aktivität, Intervention und Risikofaktoren in Zusammenhang mit Schmerzen erhoben. Bei Wissensdefiziten in diesen Bereichen besteht die Gefahr, dass veraltete Überzeugungen vermittelt werden, z. B. dass Schmerzen primär biomechanische und strukturelle Ursachen haben und daher psychosoziale und neurophysiologische Aspekte in der Therapie nicht berücksichtigt werden. Eine aktuelle Studie zeigt, dass Physiotherapeut:innen überwiegend nicht evidenzbasierte Therapien bei Patient:innen mit chronischen Rückenschmerzen auswählen [6]. Das bestätigt, dass sich Inhalte in Aus- und Fortbildung maßgeblich auf die Vermittlung von Anatomie, Physiologie und Biomechanik beschränken und ein Verständnis sowohl über die multifaktorielle Genese von Schmerz als auch über die Versorgung von Menschen mit anhaltenden Schmerzen nach aktuellen wissenschaftlichen Erkenntnissen [19] wohl nicht besteht. Diese Feststellung wird durch die Ergebnisse der vorliegenden Studie bestätigt und deutet an, dass eine Reformierung der Aus- und Fortbildung in der Physiotherapie notwendig ist. Diese Reformierung impliziert eine Akademisierung der Physiotherapie mit stetiger Vermittlung wissenschaftlicher Kompetenzen, um eine Korrektur schmerzassoziierter Überzeugungen sowie die Schließung vorhandener Lücken zwischen Wissenschaft und Praxis zu erzielen [19].

### Limitationen

Die Teilnehmer:innen wurden aus einer Physiotherapieschule in der Region rekrutiert. Somit war die Stichprobe eine nicht repräsentative Gelegenheitsstichprobe, sodass die Ergebnisse nicht auf andere Physiotherapieschüler:innen anderer Physiotherapieschulen übertragen werden können. Weiter konnte eine empfohlene Stichprobengröße von mindestens *n* = 50 für die Bestimmung der Konstruktvalidität und mindestens *n* = 100 für die interne Konsistenz nicht erreicht werden [24], was insbesondere auf die Coronapandemie zurückzuführen war. Bedacht werden muss auch, dass die für den Fragebogen zusammengestellten Items sowohl auf der Expertise und den Erfahrungen der an der Studie beteiligten Fachleute als auch auf der von ihnen identifizierten Evidenz basieren.

### Praktische Implementierung und Ausblick

In der täglichen Praxis werden Physiotherapeut:innen durch die gestellte Diagnose und in den Anamnesegesprächen mit den Patient:innen häufig mit biomechanisch und muskuloskelettal begründeten Beschwerden konfrontiert. Im „clinical reasoning“ ist es entscheidend, sich als Therapeut:in nicht ausschließlich davon leiten zu lassen. Der EKPQ soll daher als Messinstrument dienen, um zu quantifizieren, in welchem Ausmaß Physiotherapeut:innen das Schmerzproblem der Patient:innen primär postural-strukturell-biomechanisch betrachten. In dieser Studie wurde in der Zielformulierung der Begriff „Überzeugungen“ gewählt, da der Fragebogen, konform zum rNPQ [13], den Wissenstand und Überzeugungen zur Bedeutung von PSB-Faktoren im Zusammenhang mit Schmerz überprüfen soll. Es sollte jedoch nicht außer Acht gelassen werden, dass zur Versorgung von Menschen mit anhaltenden Schmerzen über das Wissen und die Überzeugungen hinaus physiotherapeutische Fertigkeiten und Kompetenzen [22] sowie Einstellungen und Verhaltensweisen [25] essenziell sind. Weitere Studien sind notwendig, um den EKPQ weiter hinsichtlich der Güte bei einer größeren Stichprobe mit Teilnehmer:innen aus verschiedenen Physiotherapieschulen und Hochschulen zu untersuchen. Auch die Items, die in der Konzipierung auf der Erfahrung und individuellen Recherche der Experten beruhen, können zukünftig, z. B. im Rahmen eines Delphi-Prozesses, überprüft und ergänzt oder ausgetauscht werden. Ferner könnte dann der EKPQ zusätzlich zum rNPQ‑D verwendet werden, um das Wissen und die Überzeugungen von Physiotherapieschüler:innen und -student:innen zu Schmerzen zu erheben und zu beurteilen. Das erlaubt möglicherweise darüber hinaus, einen Eindruck zu gewinnen, wie groß der Bedarf ist, die bestehenden Curricula der berufsfachschulischen Physiotherapieausbildung sowie der hochschulischen und universitären Studiengänge in Richtung einer evidenzbasierten Lehre über Schmerz zu überarbeiten.

## Fazit für die Praxis

- Die Physiotherapie in Deutschland erlebt derzeit einen langsamen, aber spürbaren Umbruch, in dem der Qualitäts- und Modernisierungsanspruch an die deutsche Physiotherapieausbildung die Bedeutung der Akademisierung für Physiotherapeut:innen unterstreicht.
- Rein postural-strukturell-biomechanisch (PSB)-orientierte Überzeugungen zu Schmerzen sollten durch evidenzbasierte biopsychosozial ausgerichtete Erkenntnisse ersetzt werden.
- Der in dieser Studie entwickelte Essential Knowledge of Pain Questionnaire (EKPQ) stellt einen reliablen und validen Fragebogen dar, der nach weiteren Studien und Untersuchungen zu dessen Gütekriterien als Ergänzung zur deutschen Version des Neurophysiology of Pain Questionnaire (rNPQ‑D) genutzt werden könnte, um PSB-orientierte Überzeugungen von Physiotherapeut:innen zu Schmerzen zu evaluieren.
